# Single Night Surveys of Moth Communities Can Serve as Ultra-Rapid Biodiversity Assessments

**DOI:** 10.3390/insects13121135

**Published:** 2022-12-09

**Authors:** Daniel P. Duran, Matthew Timar, Blaine Rothauser

**Affiliations:** 1 Department of Environmental Science, Rowan University, Glassboro, NJ 08210, USA; 2 Telemitra Incorporated, Haddonfield, NJ 08033, USA; 3 GZA Geoenvironmental, Inc., Fairfield, NJ 07004, USA

**Keywords:** conservation, Geometridae, Lepidoptera, Tortricidae, umbrella species

## Abstract

**Simple Summary:**

Assessing the biodiversity of a site is challenging, as a comprehensive survey of all plant and animal species is usually intractable due to limited resources. There is great interest in finding groups of organisms that may serve as proxies for the total biodiversity of a site. Numerous taxa have been proposed and utilized for rapid biodiversity assessments that allow for such a survey in a matter of weeks or less. Herein, we test the idea that nocturnal moths have many of the characteristics that make them ideal for such surveys. We demonstrate that even in a few hours of sampling during single night surveys, moth communities are predictive of regional forest types at sampling sites in New Jersey. An ordination method, known as non-metric multidimensional scaling (NMDS) analyses, revealed that moth communities differ significantly across the four forest types that we assessed. We used Analysis of Similarity (ANOSIM) R tests to quantify the degree of differentiation among moth communities, and found that the moth families Tortricidae and Geometridae predict forest communities nearly as well as the total moth diversity. Uncommon species were better predictors than common species. Host plant generalists were better predictors than specialists, which was a surprising find.

**Abstract:**

Biodiversity conservation decisions are typically based on limited data and resources. For this reason, there is great interest in surveying taxa that may allow for a rapid assessment of the biodiversity at a site. Numerous taxa have been proposed and utilized for rapid assessments that allow for such a survey in a matter of weeks or less. Herein, we test the idea that nocturnal moths have many of the characteristics that make them ideal for such surveys, such as relative ease of identification, strong ecological association with specific plant species and habitats, high alpha diversity, extended seasonal activity, and ease of trapping. We demonstrate that even in a few hours of sampling during single night surveys, moth communities are predictive of regional forest types at sampling sites in New Jersey. We sampled moths in five different forest habitats in New Jersey, USA: Pine Barrens, Upland Deciduous Forest, Palustrine Deciduous Forest, Maritime Forest, and Ruderal/Disturbed Forests, at four sites per forest type. Non-metric multidimensional scaling (NMDS) analyses revealed that moth communities differ significantly across these four forest types (*p* < 0.01). We used Analysis of Similarity (ANOSIM) R tests to quantify the degree of differentiation among moth communities, and found that Tortricidae (R = 0.657) and Geometridae (R = 0.637) predict forest communities nearly as well as the total moth diversity (R = 0.668). Uncommon species (R = 0.665) were better predictors than common species (R = 0.500). Host plant generalists (R = 0.654) were better predictors than specialists (0.538), which was a surprising find.

## 1. Introduction

A goal of most biological diversity (biodiversity) assessments is to capture a representative sample of the number of species (species richness) at a site in a practical timeframe, with limited resources. The results of biodiversity assessments can then be used to inform conservation management decisions. Assessing all of the biodiversity of a site is impractical, as a comprehensive survey of all species is intractable; thus, assessments often focus on: (1) species richness of well-studied taxonomic groups (e.g., birds, flowering plants), (2) rare species, and (3) species that are indicators of overall biodiversity and or habitat quality. These more limited foci allow for a rapid but informative assessment [1,2,3], typically completed in a matter of weeks, as opposed to a more labor-intensive survey that may require months or years.

Rapid assessments have focused on different taxonomic groups, frequently charismatic ones, including amphibians [4,5,6,7], ants [8], bats [9,10], birds [11,12], butterflies [13,14], freshwater fishes [15], katydids [16], small mammals [17,18,19], and vascular plants [20,21]. A chosen taxon and group may serve as a predictor of overall biodiversity of a site, known as an “indicator taxon” [22,23,24], or may also function as an “umbrella taxon,” a group whose conservation leads to the preservation of other taxa and ecosystems at a site [25,26,27]. When choosing a focal taxonomic group for rapid biodiversity assessments, there are several key considerations. Can the taxa be readily surveyed in the field to gain an approximation of the species richness of the target site? Is the taxonomic group sufficiently well-studied that nearly all samples can be identified to the species level relatively rapidly and at little cost? Can non-taxonomists learn to perform the identifications reliably? Will it be possible to assess the type and quality of habitat based on which species are present? 

Insects have many attributes that make them attractive for use in biodiversity assessments [28,29,30], and many groups can be sampled with relative ease using simple, inexpensive, and repeatable trapping methods, such as pitfall traps, flight-intercept traps, or light traps. For particularly well-studied or charismatic insect groups, it may be possible to identify all or nearly all samples to a species or species-group using available field guides and internet resources. An ideal focal insect group would exhibit high species richness, as indicator taxa with higher species richness outperform those with lower richness in estimating total biodiversity [31]. Moreover, it would be beneficial for a focal group to contain ecological specialists that may predict the plant community and plant species richness at a site, which, in turn, also largely dictates the animal community. Previously proposed indicator or umbrella insect groups include the following taxa, all of which are popular with amateur naturalists and professional entomologists. Tiger beetles (Cicindelidae) are a relatively small family of beetles, with 2900 species globally [32] and 120 in North America [33]. They are one of the most popular groups of insects, after butterflies and dragonflies [34], and are some of the most well-studied, non-pest arthropods. They have been studied as indicators of habitat quality, and were found to predict the biodiversity of a site, as well as the degree of degradation of tropical forests [35,36]. The longhorned beetles (Cerambycidae) have been proposed as an indicator taxon. This is a large family, with 33,000 species globally [37] and 900 in North America [38]. Some longhorned beetle species were demonstrated to be indicator species for high diversity sites in the eastern deciduous forest of the United States [39], and indicators of the quality of forest fragments in Brazilian Atlantic forest [30].

The insect order Lepidoptera is a group with extremely high species richness. More than 157,000 species of Lepidoptera have been described [40,41], with estimates reaching up to 500,000 species [42,43]. The North American fauna north of Mexico consists of at least 12,800 species parsed out across macrolepidopteran moths (55%), microlepidopteran moths (39%), and butterflies (6%) [44]. The order has been proposed as a model group that could function as an umbrella taxon [45]. They have been shown to serve as effective indicators of total biodiversity [46]. Because of the differences in sampling techniques and species diversity for the two sub-groups, we will discuss butterflies and moths separately. The butterflies (Rhopalocera) comprise a small fraction of the Lepidoptera, but are still more diverse than birds, with about 18,000 species globally and 800 found north of Mexico. Nearly all species of butterflies can be identified to a species by a trained expert, or by a non-expert with some training. Perhaps to a greater degree than any other insect group, butterflies have been recognized as umbrella taxa. For example, conservation efforts on behalf of the Karner blue butterfly (*Lycaeides melissa samuelis*) have helped to protect oak woodland savannah in North America [47]. Queen Alexandra’s birdwing (*Ornithoptera alexandrae*) has anchored efforts to protect primary forest in New Guinea [48], and the Ptunarra brown butterfly (*Oreixenica ptunarra*) is part of conservation efforts to secure and manage alpine grasslands in Tasmania [49]. 

In faunas that are relatively well known, moths are approximately fifteen times more diverse than butterflies [44]. In many temperate regions, most moths can be identified to a species or species group, or, at a minimum, a genus, by an expert or trained non-specialist. Despite the very high species richness of the group, the vast majority of species can be collected using standardizable light traps, and thus have the potential to yield high amounts of environmental data with only modest effort and expense. Moths are valuable indicator species because many exhibit dietary or ecological specialization. More than 85% of Lepidoptera are believed to specialize on one or just a set of closely related species [50,51], i.e., plants in the same genus or taxonomically proximate genera. As such, they can serve as winged proxies for a tract’s flora, and have been demonstrated to serve as effective indicators of plant diversity [52]. Moths may be better indicators of forest quality than butterflies, especially remnant late succession forests [53]. Moreover, moths are widely accepted to be sensitive indicators of disturbance and past management history [54,55]. Like butterflies, moths may also serve as umbrella taxa for specific plant communities [56]. Moth assemblages associated with pitch pine-scrub oak woodlands across the northeastern United States are used to rank stands, prioritize acquisition efforts, set management goals, guide controlled burns, and more [57,58,59]. In the United Kingdom, the straw belle (*Aspitates gilvaria*) is strictly associated with remnant calcareous grasslands, and serves as a bioindicator of this rare and declining habitat [60]. The Fabulous Green Sphinx of Kaua’i (*Tinostoma smaragditis*) is another rare moth species that may serve as a umbrella taxon for the ecosystem known in Hawaii as the diverse mesic forest [61], as it is only known from this threatened plant community [62].

Herein, we advocate for the use of night-flying moths not only as indicator or umbrella species, but specifically for use in rapid biodiversity assessments. Despite numerous detailed and longitudinal studies on moths in various ecological and conservation contexts (reviewed in [56]), moths have been underutilized in rapid surveys of general biodiversity (however, see [63,64,65]). Assessing the moth community at a site with only one evening of sampling can produce an ultra-rapid biodiversity assessment (URBA). To test whether moth communities could be predictive of the overall plant community, we performed non-metric multidimensional scaling analyses of moth samples from five forest types in New Jersey, USA.

## 2. Materials and Methods

### 2.1. Sampling Methods

Moths were sampled using a 1000 W metal halide full-spectrum light and an ultraviolet 15 W blacklight next to a white canvas tarp. Specimens were not collected, but were photo-sampled. At each sampling event, lights were run for a single night in the summer during the peak flight period, June 25 to August 15. Lights were kept on from dusk until 2 a.m. Surveys were not conducted on nights when it rained >0.5” during the day, as previous experience had shown that this reduced nocturnal moth activity.

To facilitate identifications, high-quality photos were taken of moths at rest on the white canvas sheet using a Canon 5D Mark IV camera, with a 100 mm macro EF L series lens. Moths were photographed both dorsally and laterally when possible. All moths were identified to a species or species group using photo vouchers, either during the sampling event or at a later time. Identifications were performed by non-specialists using Beadle and Leckie [66] and Moth Photographer’s Group: (https://mothphotographersgroup.msstate.edu/ accessed on 25 June 2015–27 July 2020). The time spent in species identifications (post-sampling) ranged from 2–4 h per site. All moth data can be found in Supplementary Table S1.

### 2.2. Forest Types

**Figure 1:**
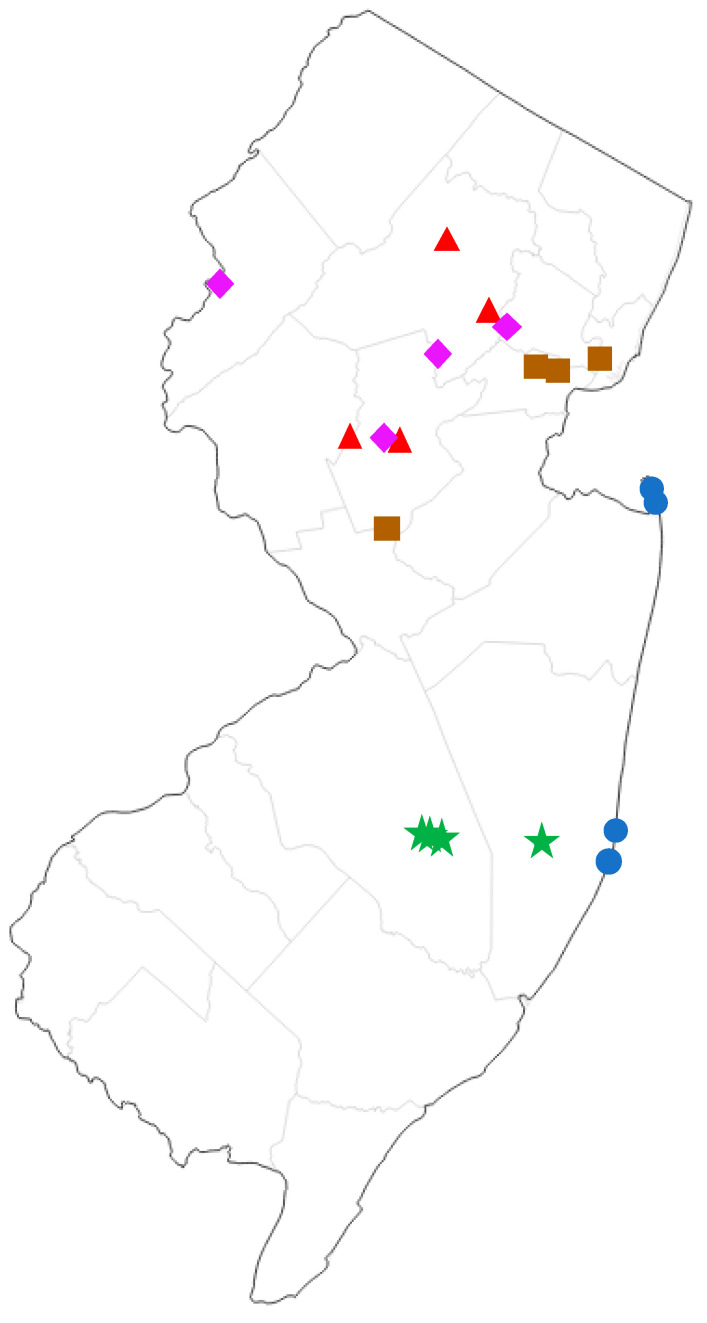
Map of sampling sites in New Jersey, USA. Green stars = pine barrens, blue circles = maritime forest, purple diamonds = palustrine deciduous forest, red triangles = upland deciduous forest, brown squares = ruderal (highly disturbed).

We compared the moth communities of five different forest types found in New Jersey: Pine Barrens, Maritime Forest, Upland Deciduous Forest, Palustrine/Lowland Deciduous Forest, and Disturbed/Ruderal. The following are representative plant species that characterize each forest type. Pine Barrens: pitch pine (*Pinus rigida*), scrub oak (*Quercus ilicifolia*), scarlet oak (*Q. coccinea*), white oak (*Q. alba*), blackjack oak (*Q. marilandica*), black gum (*Nyssa sylvatica*), Atlantic white cedar (*Chamaecyparis thyoides*), dangleberry (*Gaylussacia frondosa*), highbush blueberry (*Vaccinium corymbosum*), lowbush blueberry (*V. angustifolium*), bracken fern (*Pteris aquilina*); Maritime Forest: eastern red cedar (*Juniperus virginiana*), sassafras (*Sassafras albidum*), swamp white oak (*Q. bicolor*), red oak (*Q. rubra*), pitch pine (*Pi. rigida*), American holly (*Ilex opaca*), red maple (*Acer rubra*), hackberry (*Celtis occidentalis*), beach plum (*Prunus maritima*), pasture rose (*Rosa carolina*), poison ivy (*Toxicodendron radicans*), Virginia creeper (*Parthenocissus quinquefolia*), porcelain berry (*Ampelopsis brevipedunculata*), multiflora rose (*Rosa multiflora*), Japanese honeysuckle (*Lonicera japonica*), American beachgrass (*Ammophila breviligulata*); upland deciduous forest: chestnut oak (*Q. montana*), red oak (*Q. rubra*), black oak (*Q. velutina*), tulip tree (*Liriodendron tulipifera*), sugar maple (*Ac. saccharum*), red maple (*Ac. rubra*), black birch (*Betula lenta*), shagbark hickory (*Carya ovata*), pignut hickory (*Ca. glabra*), persimmon (*Diospyros virginiana*), sweetgum (*Liquidambar styraciflua*), white ash (*Fraxinus americana*), black walnut (*Juglans nigra*), white pine (*Pi. strobus*), eastern red cedar (*J. virginiana*), American holly (*I. opaca*); Palustrine deciduous forest: silver maple (*Ac. saccharinum*), red maple (*Ac. rubra*), shagbark hickory (*Ca. ovata*), pignut hickory (*Ca. glabra*), American sycamore (*Platanus occidentalis*), boxelder (*Ac. negundo*), pin oak (*Q. palustris*), red oak (*Q. rubra*), speckled alder (*Alnus incana*), autumn olive (*Elaeagnus umbellata*), sweetgum (*Liq. styraciflua*), common elderberry (*Sambucus canadensis*), tulip tree (*Lir. tulipifera*), green ash (*Fraxinus pennsylvanica*); Ruderal: tree-of-heaven (*Ailanthus altissima*), autumn olive (*E. umbellata*), Callery pear (*Pyrus calleryana*), northern catalpa (*Catalpa speciosa*), honey locust (*Gleditsia triacanthos*), black locust (*Robinia pseudoacacia*), Oriental bittersweet (*Celastrus orbiculatus*), multiflora rose (*Ros. multiflora*), Japanese honeysuckle (*Lo. japonica*), red maple (*Ac. rubra*), Chinese bush clover (*Lespedeza cuneata*), porcelain berry (*Am. brevipedunculata*). Surveys were conducted at four geographic sampling locations for each forest type (Figure 1, detailed locality data in Supplementary Table S2).

### 2.3. Statistical Analyses of Moth Communities

We used Non-metric Multi-Dimensional Scaling (NMDS) analysis, an ordination method, to cluster sampling localities by the overall similarity of their species communities in two-dimensional space. All analyses were run in R 4.0.3 [67] using the vegan package. Bray–Curtis Index [68] calculations were used to create the distance matrix used in the NMDS. Unlike Euclidean distances, Bray–Curtis distances are not sensitive to total abundances, and are well-suited for most ecological comparisons. Bray–Curtis distances are more appropriate than Euclidean distances when much of the data in the matrix consists of zeros, as was the case with our moth species counts.

For each iteration of the NMDS, a stress value was calculated, which was a measure of disagreement between observed and fitted distances. Stress can be thought of as a value that represents the difference between the distances in the reduced dimensional space compared to the full multidimensional space. Conceptually, stress is roughly equivalent to a measure of “goodness of fit” to the NMDS ordination. Generally, as a rule of thumb, stress values of <0.1 are considered a very good fit, <0.2 are considered a good fit, and those approaching 0.3 are a poor fit.

**Figure 2:**
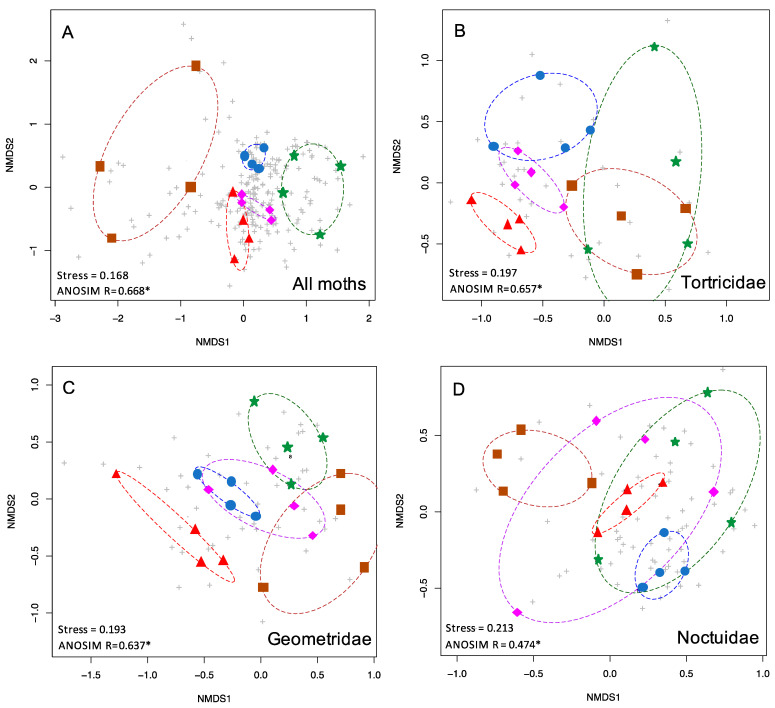
nMDS plots showing the two-dimensional ordination for moth communities in five habitat types. Colored symbols are as in Figure 1. Grey pluses indicate individual species. Shown here are (**A**) the complete moth community (510 spp.), (**B**) Tortricidae (54 spp.), (**C**) Geometridae (56 spp.), and (**D**) Noctuidae (124 spp.).

**Figure 3:**
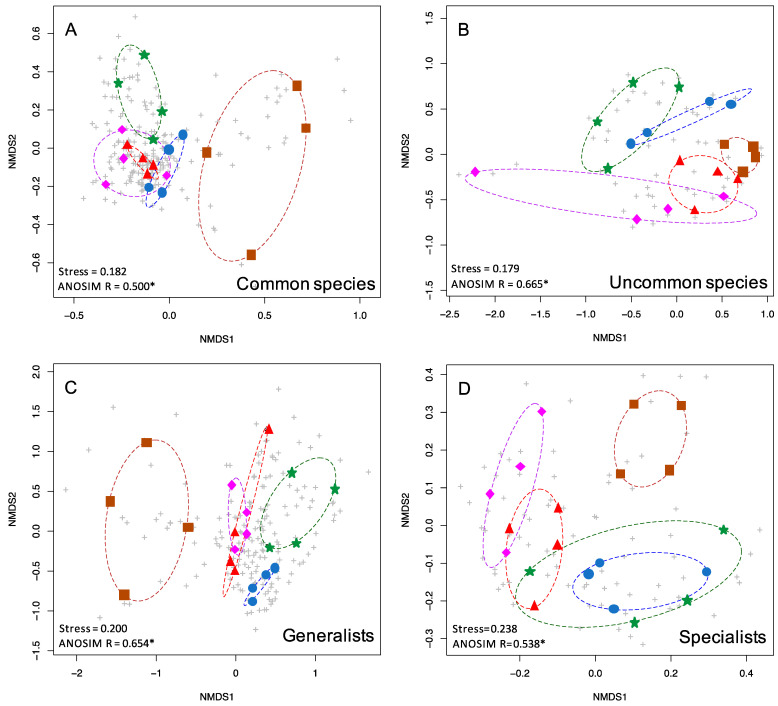
nMDS plots showing the two-dimensional ordination for moth communities in five habitat types. Colored symbols are as in Figure 1. Grey pluses indicate individual species. Shown here are (**A**) common species (363 spp.), (**B**) uncommon and rare species (147 spp.), (**C**) generalists (325 spp.), and (**D**) specialists (123 spp.).

To graphically illustrate the clustering of communities of each forest type, the ordiellipse function within the vegan package was used, implemented in R 4.0.3. This function generated convex hulls connecting the communities of each treatment (forest type) (Figure 2 and Figure 3). 

Analysis of Similarity (ANOSIM) was performed to determine whether there was a statistical difference between the communities of two groups of samples. In addition, the ANOSIM R statistic compares the mean of ranked dissimilarities between groups to the mean of ranked dissimilarities within groups. Values range from 0 (identical) to 1 (completely dissimilar). 

We performed the NMDS and ANOSIM analyses on the total moth dataset as well as on other taxonomically and ecologically interesting subsets. We also examined how well the five most speciose moth families predicted forest types. Additional comparisons included common vs. uncommon species (based on [69]) and specialists (larvae feed on two genera of plants or fewer) vs. generalists (larvae feed on three or more genera of plants). Because the definitions of specialist and generalist could affect the outcome of the results, we also ran the NMDS and ANOSIM analyses using relaxed criteria for feeding specialization, with specialists defined as larvae that feed on one family of plants and generalists on two or more [70].

## 3. Results

When all 510 species of moths were included in the NMDS analysis, the five forest types were found to be significantly different in species composition from each other (*p* < 0.001) (Table 1, Figure 2A). Stress was 0.168, indicating that the NMDS ordination was a good fit. For this full dataset, the ANOSIM R statistic was 0.668, indicating considerable differentiation between the moth communities in different forest types, and a greater degree of differentiation than any subset of the data (Table 1).

The five most speciose moth families, Noctuidae, Erebidae, Crambidae, Geometridae, and Tortricidae, were analyzed individually, and each displayed significant differences in species composition by forest types (all *p* < 0.001, Table 1). NMDS stress values were under 0.2 for Tortricids and Geometrids (0.197, 0.193) and above 0.2 for Crambids, Erebids, and Noctuids (0.2, 0.211, 0.213). Tortricids and Geometrids displayed the most community structuring based on the highest ANOSIM R values, greater than 0.6, (R = 0.657, 0.637) which was similar to the full dataset (0.668), whereas Crambids, Erebids, and Noctuids exhibited lower ANOSIM R values (R = 0.579, 0.562, 0.474). Representative NMDS plots are shown in Figure 2.

Uncommon and common moths displayed low stress values (0.179, 0.182), with uncommon species exhibiting greater differentiation between moth communities in different forest types based on ANOSIM R values compared to common species (0.665, 0.500). Both dietary generalists and specialist species displayed stress values over 0.2 (0.2, 0.238). Generalists (either definition, see Materials and Methods) exhibited greater moth community differentiation compared to specialists, based on ANOSIM R values (0.654, 0.538 for primary definition, 0.621, 0.544 for alternate definition).

## 4. Discussion

Moths are highly diverse, globally distributed, and their ecological significance cannot be overstated, as they are a critical part of terrestrial food webs [71]. They are often specialists in their diet breadth or habitat requirements [50,51], and, accordingly, moths have been proposed as umbrella and indicator taxa [56]. Still, they have been underutilized as a focal taxon for rapid biodiversity assessments (however, see [63,64,65]). 

Based on the results of our NMDS and ANOSIM analyses (Table 1; Figure 2 and Figure 3), moth communities show significant differentiation between different forest types, even within a relatively small regional area (New Jersey, Figure 1). Moreover, each sampling event was conducted over several hours in a single evening, underscoring how rapid this method is while still providing sufficient data to detect such patterns. Based on these attributes, moths are well-supported as a potential umbrella taxon, and can serve as a valuable focal group for biodiversity assessments. Given the minimal time requirements and relative ease of sampling, we refer to the type of moth surveys described herein as ultra-rapid biodiversity assessments (URBA). Typical RBAs surveys take on the order of weeks. 

The total dataset (n = 510 species, 20 sites, five forest types) showed strong community structuring, with moth communities clustering by forest type and largely non-overlapping (Figure 2A). Moreover, our NMDS and ANOSIM analyses also demonstrated that some families of moths serve as more satisfactory proxies for the total moth diversity, and can differentiate the forest types (Figure 2B,C). Thus, these taxa could eliminate the need for identifying hundreds of species of moths at a site. For example, both Tortricids and Geometrids were highly predictive of forest type on their own (*p* < 0.001 for both; ANOSIM R = 0.657, 0.637), although both showed some overlap of the moth communities within different forest types. However, we caution that several lineages of Tortricids are challenging to identify to species. Conversely, Geometrids may be particularly valuable as indicators, as these are relatively easy to identify, although a few require genitalic examination to unequivocally identify to species. They were the fourth most diverse family of moths in our dataset (n = 56 species), yet they predicted forest types better than the much more diverse Erebidae (n = 83 species, R = 0.562) and Noctuidae (n = 124 species, R = 0.475). One possible explanation for their predictive power could be that many Geometrids tend to be weak flyers, and are thus less likely to occur out of habitat (pers. comm. D. Wagner). The dearth of Geometridae in Ferguson’s [72] list of long-distance lepidopteran dispersers underscores this point. The vast majority of long-distance dispersing moths are Erebids and Noctuids (among which are many crop pests). Regardless of taxon, dispersive taxa create “noise” in analyses seeking to characterize the uniqueness of a given habitat patch.

Uncommon species of various taxa are frequently used in biodiversity assessments [1,2,3]. We found this to be true in our study, i.e., they showed the greatest degree of community structuring of any subset of the data. Uncommon moths were nearly as differentiated by forest type (R = 0.665) as all moths (R = 0.668) despite being based on only 147 species instead of the total 510 species. One unexpected finding was that host plant generalist species (those that feed on two or more genera of plants) were better predictors of forest type (R = 0.654) than host plant specialists (R = 0.538). One would predict that specialists should be better predictors of forest type, but it may be that generalist feeders are not oriented towards specific host plants, but rather that they may be associated with particular habitats or forest types. 

In conclusion, our study demonstrates that moth communities are significantly different in different forest types within a small geographic area (New Jersey), as shown by NMDS and ANOSIM analyses. These findings support the use of moths as umbrella taxa. Our moth surveys were conducted over several hours at each site, and this underscores how rapidly information can be collected, permitting ultra-rapid biodiversity assessments (URBA). Two moth families, Geometrids and Tortricids, were better predictors of forest type than others, and these were not simply the most speciose families. Future studies should focus on whether moth communities can predict habitat quality, not just habitat type. If so, moth surveys would take on even greater utility for conservation planning. 

## Figures and Tables

**Table 1:** Statistical values for nMDS and ANOSIM analyses.

Taxon	N of Taxa	ANOSIM R	*p* Value <	NMDS Stress
All moths	510	0.668	0.001	0.168
Tortricidae	54	0.657	0.001	0.197
Geometridae	56	0.637	0.001	0.193
Crambidae	67	0.579	0.001	0.200
Erebidae	83	0.562	0.001	0.211
Noctuidae	124	0.475	0.001	0.213
Uncommon moths	147	0.665	0.001	0.179
Common moths	363	0.500	0.001	0.182
Generalists	325	0.654	0.001	0.200
Specialists	123	0.538	0.001	0.238

## Data Availability

All data collected in this study are publicly available and exist in the supplementary materials above.

## Supplementary Materials

The following supporting information can be downloaded at: https://www.mdpi.com/article/10.3390/insects13121135/s1, Table S1: Moth sampling data. Table S2: Detailed locality data.
